# Measuring Adherence Within a Self-Guided Online Intervention for Depression and Anxiety: Secondary Analyses of a Randomized Controlled Trial

**DOI:** 10.2196/30754

**Published:** 2022-03-28

**Authors:** Maria Hanano, Leslie Rith-Najarian, Meredith Boyd, Denise Chavira

**Affiliations:** 1 Department of Psychology University of California, Los Angeles Los Angeles, CA United States; 2 Department of Psychology Harvard University Cambridge, MA United States; 3 Dean of Student's Office Harvard University Cambridge, MA United States

**Keywords:** self-guided, adherence, depression, anxiety, online intervention

## Abstract

**Background:**

Self-guided online interventions offer users the ability to participate in an intervention at their own pace and address some traditional service barriers (eg, attending in-person appointments, cost). However, these interventions suffer from high dropout rates, and current literature provides little guidance for defining and measuring online intervention adherence as it relates to clinical outcomes.

**Objective:**

This study aims to develop and test multiple measures of adherence to a specific self-guided online intervention, as guided by best practices from the literature.

**Methods:**

We conducted secondary analyses on data from a randomized controlled trial of an 8-week online cognitive behavioral program that targets depression and anxiety in college students. We defined multiple behavioral and attitudinal adherence measures at varying levels of effort (ie, low, moderate, and high). Linear regressions were run with adherence terms predicting improvement in the primary outcome measure, the 21-item Depression, Anxiety, and Stress Scale (DASS-21).

**Results:**

Of the 947 participants, 747 initiated any activity and 449 provided posttest data. Results from the intent-to-treat sample indicated that high level of effort for behavioral adherence significantly predicted symptom change (F4,746=17.18, *P*<.001; and β=–.26, *P*=.04). Moderate level of effort for attitudinal adherence also significantly predicted symptom change (F4,746=17.25, *P*<.001; and β=–.36, *P*=.03). Results differed in the initiators-only sample, such that none of the adherence measures significantly predicted symptom change (*P*=.09-.27).

**Conclusions:**

Our findings highlight the differential results of dose-response models testing adherence measures in predicting clinical outcomes. We summarize recommendations that might provide helpful guidance to future researchers and intervention developers aiming to investigate online intervention adherence.

**Trial Registration:**

ClinicalTrials.gov NCT04361045; https://clinicaltrials.gov/ct2/show/NCT04361045

## Introduction

There has been a proliferation of online interventions aimed at preventing and treating mental health disorders (eg, [1]). Online interventions have the potential to reach a wide audience while bypassing barriers that are more common to traditional face-to-face interventions, such as financial cost, inaccessibility, and stigma [2]. The flexible nature of online interventions provides its users autonomy to interact with content according to their unique schedule and preferences [3]. Self-guided online interventions, in particular, maintain anonymity, address some concerns related to stigma, are less costly, and require little time from mental health professionals [4,5]. Recent meta-analyses have shown that both guided and self-guided online interventions can be effective for treating a range of problems such as depression and anxiety [1,3,6]. Moreover, research has shown that online interventions attract a large number of individuals (eg, 38,000 registrants to MoodGYM) who experience significant mental health symptoms [2].

Despite the promising nature of self-guided online interventions, multiple reviews report high dropout and poor adherence rates [1,7]. A meta-analysis found that while 72% of adults adhered to guided online interventions (ie, guided by a mental health professional), only 26% adhered to self-guided online interventions [8]. As one example, an online self-guided and publicly available cognitive behavioral therapy program aimed at preventing depression and anxiety attracted 38,000 registrants, but only 3.9% adhered to the intervention (adherence was defined as completing 3 of the 5 modules; [2]). Eysenbach [9] described this “law of attrition” as a fundamental challenge for online intervention trials—relative to drug or psychosocial therapeutic trials—because participants are less closely supervised and thus, they receive more sporadic doses of an intervention, or even none at all. Because of the variability in adherence rates, it is difficult to measure and make conclusions about the effectiveness of such interventions. Presently, there is limited understanding about how adherence within self-guided online interventions affects clinical outcomes, which in turn limits our ability to identify those intervention components that might be the necessary mechanisms of change.

There are currently many challenges to understanding adherence and its relation to outcomes within online interventions. Such challenges include varying ways of operationalizing and measuring adherence, which reduce our ability to compare adherence rates across various trials [10,11]. The term “adherence” is often used interchangeably with terms such as engagement, user retention, or dropout (eg, [12]). Focusing on definitions for “adherence,” Sieverink and colleagues [11] analyzed how 62 studies operationalized adherence to online interventions (both guided and self-guided), and found that operationalizations fell into 3 categories: (1) “the more usage, the better”; (2) researcher-defined “intended use” but without justification (eg, a user is adherent when logging in at least once a week for 3 weeks); and (3) researcher-defined “intended use” justified using theory, evidence, or rationale (eg, We know from previous research that users benefit the most from the technology when finishing module 4, so a user is adherent once module 4 is completed). Beintner and colleagues [10] found that an array of usage measures can define adherence, such as percentage of participants completing all modules, percentage of participants completing each module, percentage of participants who visited the website, average number of log-ins, and average duration of visit. This variability in measurement has prevented convergence of evidence on which adherence measures are valid. Consequently, the lack of standardized adherence measures perpetuates a cycle where researchers use a wide variety of adherence measures for self-guided online interventions. The result is less clarity on a conceptual framework of adherence as it applies to online interventions.

Various reviews provide actionable recommendations for improving the standardization of how we define, measure, and report adherence to online interventions (eg, [10,11]). We have distilled various recommendations into 2 broad topics we believe to be particularly useful and feasible: (1) creating and reporting multiple measures, and (2) relating these measures to outcomes.

The first recommendation—to create and report multiple measures of adherence—facilitates our understanding of the multiple ways by which participants may adhere to online interventions [10]. According to Beintner and colleagues [10], such measures should be both universal (eg, measures of average number of completed sessions) and intervention specific (eg, completing diaries or discussion boards) [10]. Universal measures are most frequently used, allowing for the comparison of such metrics across studies. Measures of adherence specific to the intervention should also be created and reported, in order for study designers to understand whether study-specific components are beneficial. It is also recommended that measures reflect the interventions’ intended use [11]. This recommendation is in line with the World Health Organization’s definition of adherence as “the extent to which a person’s behavior – taking medication, following a diet, and/or executing lifestyle changes – corresponds with agreed recommendations from a health care provider” [13]. Sieverink and colleagues [11] propose that it is most useful to understand the threshold required, or how much adherence is necessary, in order for it to predict improved outcomes, rather than assuming that more adherence is always better. In summary, it may be advantageous to have some universal adherence measures and some intervention-specific adherence measures based on the intervention’s intended/recommended minimum use.

The second recommendation is to test the relationship between measures of adherence and clinical outcomes to inform the validity and utility of adherence measures. This process is critical to make correct interpretations about the impact of adherence on target outcomes and the effectiveness of the online intervention [10,14]. A recent review found that treatment adherence (defined as the total number of sessions completed by the participant divided by the total number of treatment sessions) was significantly related to outcomes within self-guided online interventions [4]. Another study, which examined multiple types of adherence measures (activities completed per login, total time spent online, total time spent online per log-in, combined modules, and activities measure), found that only the number of activities completed per login was significantly associated with better outcomes for those who received the online intervention [15]. They also divided patterns of usage into 3 levels (low, medium, and high) and found that medium-level users did not differentially benefit from the intervention compared with low-level users [15]. In turn, measures of adherence that are predictive of symptom improvement should inform an online intervention’s engagement strategies.

In this study, we follow these recommendations to test questions about *how much* adherence and *which* measures of adherence matter in predicting symptom improvement within a self-guided online intervention. The first aim of the study is to demonstrate an example of the process of testing multiple measures as applied to a specific self-guided online intervention. The second aim is to translate the results of this process into recommendations for future researchers and interventionists to consider when making decisions about creating and testing measures of adherence in online interventions.

## Methods

### Design

We conducted a secondary analysis using data from a randomized controlled trial (RCT) that tested a self-guided web-based mental health skills program for universal prevention of anxiety and depression in university students [16]. Primary results of this trial showed small intervention effects overall [16]. Participants were randomly assigned to an immediate intervention condition, (ie, they could access the intervention upon signing up), or a delayed access condition (ie, they were on a waitlist initially and only granted access to the online platform after the immediate intervention condition was over). The timing of both conditions was staggered such that the start and finish week of the intervention access for both conditions corresponded to equivalent weeks within the respective academic quarter. For the purposes of this study, data collected from both conditions were collapsed such that pre-/postscores reflect each participant’s status immediately before receiving the intervention and immediately after receiving the intervention.

### Participants

Participants were at least 18 years old, undergraduate and graduate students at the University of California, Los Angeles. Recruitment efforts included, but were not limited to, department-wide emails, flyers posted around the university, social media, and announcements in psychology courses. Compensation for research survey participation included entry into US $10-US $100 gift card drawings or course credit. Exclusion criteria were being enrolled in a similar anxiety and depression treatment study, invalid data reporting (eg, straight-lining or high inconsistency in responses), and not verifying one’s online intervention account or not completing the account setup process. Out of a total of 947 participants, 747 initiated any activity, and 449 had posttest outcome data.

### Intervention

A more detailed description of the intervention and screenshots of the platform can be found in the primary intervention [16]. In this section, we describe the information most relevant for understanding adherence within the context of the tested intervention. The program consisted of 8 weeks, each of which focused on an evidence-based skills theme. Participants were allowed to choose what they would like to practice from a list of activities relevant to the respective weeks’ theme. For example, the “Change Your Thinking” week provided instructions for cognitive restructuring strategies, and activities that participants could practice and log included “identify any unhelpful thinking patterns,” “identify evidence for and against the unhelpful thought,” “shift your attention,” etc. As another example, the “Pause” week focused on strategies to foster mindfulness practice in daily life, and provided instructions for activities such as “eat mindfully,” “listen mindfully,” “meditate mindfully.” With regard to how many skills participants would practice each week, there were no such requirements in the instructions of the intervention, but rather the online platform used a virtual medal system to incentivize more practice. Participants were awarded medals depending on the amount of activities logged each week: “Bronze” is awarded when a participant completes at least one log for that week; “Silver” is awarded when a participant completes three logs for that week; “Gold” is awarded when a participant completes at least five logs for that week. Finally, in addition to logging any skills they practiced, participants were prompted to submit an end-of-week check-in comprising 2 reflective questions about skills practiced that week. Examples of reflective questions that participants could answer during weekly check-ins were as follows: Week 3: “Which technique was most helpful for you?”; “Did this week move you closer or not to your goals?”. Week 7: “Did being more mindful make your more aware of anything in your life or daily experiences?”; “Did this week move you closer or not to your Life 2.0?” Submitting a check-in was completely optional, however, they are required to log any activities they practice on the activity log tab on the platform.

### Measures

#### Adherence

Following the first recommendation, multiple measures of adherence were created that were (1) both universal and study specific and (2) designed to capture the intervention’s intended use.

Staudt’s model of adherence (referred to as *engagement* in Staudt 2007 [17]) informed the selection of our universal measures. Specifically, Staudt proposes that adherence can be thought of universally as involving behavioral and attitudinal aspects. This model defines behavioral adherence as, “client performance of the tasks that are necessary to implement treatment and to ultimately achieve outcomes.” Examples of behavioral adherence in face-to-face interventions include maintenance of appointments, homework completion, and responsiveness to the practitioner. The model defines attitudinal adherence as, “the emotional investment in and commitment to treatment that follow from believing that it is worthwhile and beneficial” [17]. Examples of attitudinal adherence in face-to-face interventions include positive attitude toward the intervention, perceiving the intervention as worthy of time and energy, perceiving the benefits outweigh the costs of the treatment. The author explains that a person’s attitude toward the intervention represents the “heart” of adherence and is necessary for participants to make meaningful changes during an intervention. In the context of our study, to ensure that study-specific behavioral and attitudinal adherence measures were defined based on the interventions’ intended use, we referred to the intervention’s weekly instructions provided to participants. The resulting adherence measures are described in more detail below and summarized in Table 1.

**Table 1:** Definition of 6 measures of adherence based on recommendations.

Measure	Behavioral adherence^a,b^	Attitudinal adherence^c,d^
Minimal effort	Number of weeks with at least one skill practice log	Number of weeks with check-in word count of any length
Moderate effort	Number of weeks with at least three skill practice logs	Number of weeks with check-in word count ≥ respective average
High effort	Number of weeks with at least five logs	Number of weeks with check-in wordcount ≥ respective average +1 SD

^a^Universal definition: performance of intervention-related tasks.

^b^Intervention-specific definition: practice of skills, per amount of weekly logged activity within user account.

^c^Universal definition: emotional investment.

^d^Intervention-specific definition: elaborateness of written responses to reflection prompts, per word count of weekly check-ins within user account.

#### Behavioral Adherence

Regarding behavioral adherence, as a reminder, participants practiced skills each week and were awarded 1 of 3 medals depending on the number of skills practiced (see the “Intervention” section; [16]). As such, behavioral adherence was operationalized by the skills practice logs within each user’s account. We created 3 behavioral adherence measures, which reflected the number of weeks with behavioral adherence at 3 effort levels (ie, minimal, moderate, and high), categorized according to the medal system: (1) Minimal behavioral adherence: number of weeks with at least one skills practice log; (2) Moderate behavioral adherence: number of weeks with at least three logs; and (3) High behavioral adherence: number of weeks with at least five logs.

#### Attitudinal Adherence

In order to have somewhat parallel operationalizations of behavioral and attitudinal measures, we also applied the 3 levels of effort to attitudinal adherence. For attitudinal adherence, participants were prompted to respond to 2 weekly check-in questions, though they were not instructed on how much to write. As such, attitudinal adherence was operationalized as the extensiveness of user’s open-ended reflective responses on weekly check-ins. Because these check-ins are optional and encourage the participants to reflect on their experience and growth, we believed that the act of electing to complete a check-in would reflect the participant’s emotional investment in the intervention. Moreover, writing a longer response to an optional check-in question, rather than briefly answering the question, reflects varied levels of adherence effort by participants. To create minimal, moderate, and high levels of attitudinal adherence, we used word count on weekly check-ins. One previous study found that diary entry word count in an online intervention was correlated with the number of activities that the individual logged [18]. First, we cleaned weekly check-ins to remove (1) duplicate responses, and (2) random or nonalphanumeric characters. Second, we obtained means of word count on end-of-week check-ins for each week and used them as cut-offs determining each level of effort. Third, we created the attitudinal adherence measures at each effort level, defined as

- Minimal: number of weeks with at least one word.
- Moderate: number of weeks with word mean at mean or above for each respective week.
- High level: number of weeks with word mean at 1 SD from mean and above for each respective week.

For example, the mean word count on the third week of the intervention was 32.87; participants at or above this mean for the third week were considered to have moderate attitudinal adherence to the intervention for that week.

### Primary Outcome: Depression, Anxiety, and Stress

The primary outcome measure in this study was the 21-item Depression, Anxiety, and Stress Scale (DASS-21), which assesses self-reported symptoms of depression, anxiety, and stress [19]. In previous studies the measure had demonstrated adequate internal consistency (Cronbach α=0.83-0.90) and construct validity [19]. In this RCT, internal consistency of the DASS-21 was adequate (total: Cronbach α=.92; depression: Cronbach α=.89; anxiety: Cronbach α=.79; stress: Cronbach α=.82) [16]. DASS-21 total symptom change scores were calculated (post-pre scores) and used as the primary outcome. We expected use of DASS-21 total scores (as opposed to subscale scores, for example) to maximize the power of our analyses, given that it showed the largest effect size per our primary intervention main effect analyses [16] (Exploratory linear regression analyses served to support that there were indeed decreased strength and significance in the relationships between adherence variables and DASS-21 subscales.).

### Covariates

Covariates selected for the study included (1) condition (intervention upon signing up vs delayed access condition); (2) suicidal ideation at baseline measured through the use of question 9 on the 9-item Patient Health Questionnaire (PHQ-9) [20]; and (3) gender. The PHQ-9 is a 9-question measure widely used to assess self-reported symptoms of depression. We operationalized suicidal ideation through question 9, which directly assesses for suicidal ideation [20]. Gender was defined as binary “female” or “male,” based on the demographics data linked from participants official student records. The rationale for examining suicidal ideation and gender as covariates is outlined in the primary RCT [16]. Inclusion of these 3 covariates was thus consistent with those tested in the primary online intervention analyses. These 3 covariate variables were also included as auxiliary variables informing our multiple imputation model.

### Other Measures

Remaining variables were included only as auxiliary variables for the multiple imputation model in predicting missing data in our primary outcome variable (see the “Data Analysis” section for more details). The Grit Scale (GRIT) is a 12-item measure aimed at measuring traits of perseverance, maintaining focus, and interest in long-term goals [21]. The Treatment Motivation Questionnaire (TMQ) [22] assesses reasons for initiating and remaining in treatment; we used an adapted version of this measure to apply to the tested online intervention [16]. The Subjective Happiness Scale (SHS) [23] is a 4-tem scale that measures global subjective happiness.

### Statistical Analysis

#### Preliminary Analyses

First, given the amount of missing outcome data at posttest, we conducted a series of independent unpaired *t* tests and chi-square tests to determine whether any variables were significantly related to posttest missing data status, which would suggest if data were not missing completely at random (MCAR). Relatedly, to support our selection of the multiple imputation model to deal with missingness, we aimed to verify that our data set was not MCAR using the Little MCAR test. Second, to identify necessary covariates for the regression analyses, we conducted the same series of analyses but this time to assess all variables as predictors for significant differences in DASS-21 change scores. Third, we conducted linear regression assumption checks to determine if (1) the necessary conditions were met, and (2) conducting multiple linear regressions with more than 1 adherence predictor simultaneously was appropriate [24].

#### Multiple Imputation

Given the degree of missing DASS-21 outcome data at posttest (370/947, 39.1%) and results of preliminary analyses (reported below), we determined that analyzing data only from complete cases would likely produce biased findings with decreased power. As such, we implemented multiple imputation procedures for missing posttest DASS-21 values (ie, outcome), predicted by auxiliary variables: the 6 measures of adherence, condition, gender, suicidal ideation, GRIT, TMQ, PHQ-9, SHS, and total baseline DASS-21. We were liberal in our selection of auxiliary variables, and included all measures collected at baseline in the imputation model to preserve any complex associations that may exist among the variables, especially considering that adding too many variables is unlikely to produce bias [25]. We also elected to run 50 imputations to decrease standard errors and produce stable estimates, which require between 50 and 100 imputations [25].

#### Linear Regressions

Multiple linear regressions were run including all identified covariates in step 1, and then adding the respective adherence measure as a variable in step 2, predicting the main outcome variable of DASS-21 change scores (posttest – baseline scores). Results from the 50 multiple imputations were then pooled and analyzed. Given that Type II error rates increase as more models are tested, we calculated Benjamini-Hochberg critical values [26], an alternative to simply applying a *P* value of .05 across all models.

### Ethics Approval

This study was approved by the appropriate University of California Los Angeles Institutional Review board (IRB# 17-000761).

## Results

### Participants

The sample consisted of 947 students, with a mean age of 23.01 (SD 5.56), with 729 (77.0%) identified as female, 299 (31.6%) as White, and 121 (12.8%) as international. The groups that received the intervention immediately consisted of 587 participants, and the delayed intervention group consisted of 360 participants. The number of program initiators in this sample, that is, those with any activity stored within their user account, was 747 students. Baseline DASS-21 total scores had a mean of 17.30 (SD 10.47), and the mean DASS-21 change was –2.67 (SD 9.38). The number of participants with posttest outcome data (DASS-21) was 449/947 (47.4% of sample).

### Adherence

**Figure 1:**
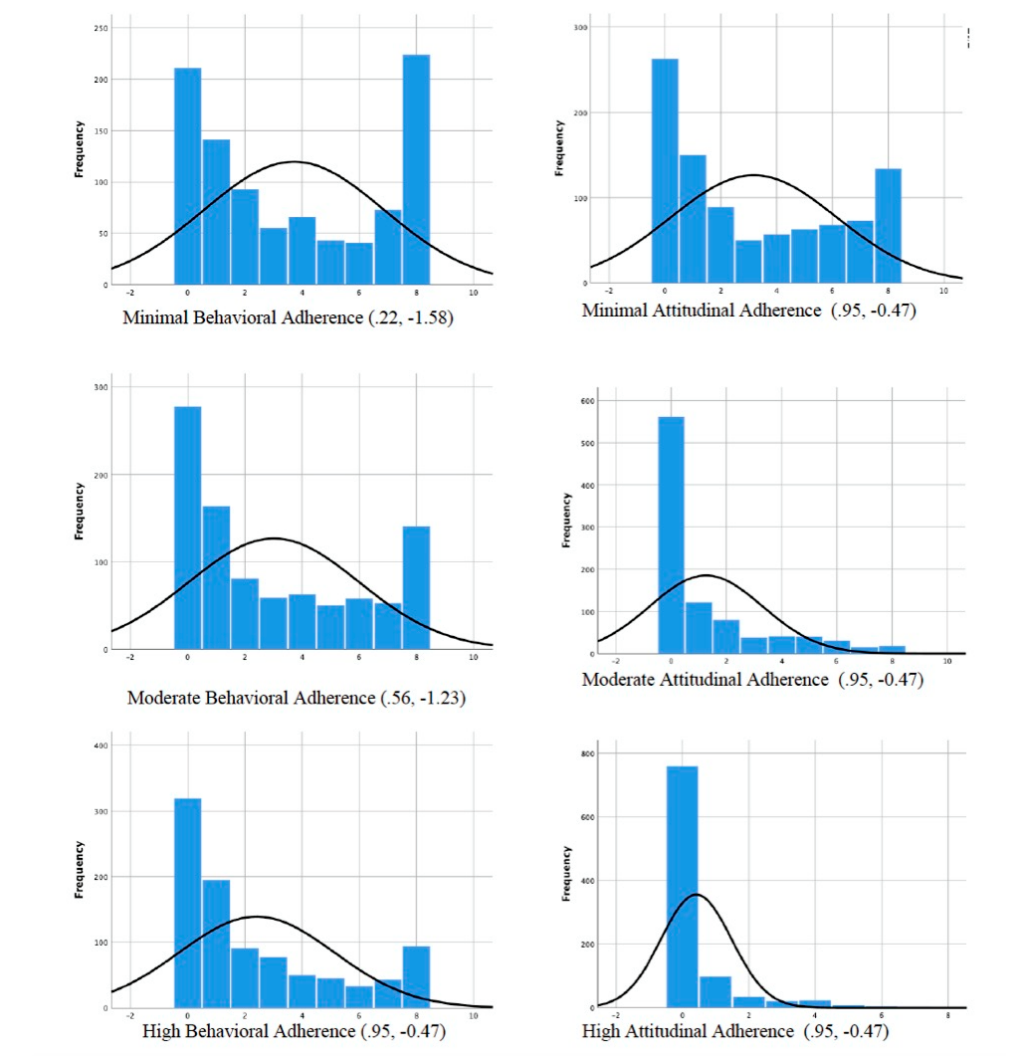
Distribution plots of each adherence measure (Adherence measure, Skewness, Kutorsis).

For behavioral adherence, the mean number of skills practice logs per module overall was 2.14 (n=947). The percentage of 0 skills practice logs on each module ranged from 47.7% (452/947) to 63% (597/947). When participants with 0 logs on each separate module were excluded, the mean number of skills practice logs was 4.51. The module with the highest average skills practice logs was the Welcome module (mean 5.06 [SD] 2.25), and the one with the lowest was the Physical Exercise module (mean 3.58 [SD] 1.92). For attitudinal adherence, the mean weekly check-in word count for modules overall was 57.70. The module with the highest check-in word count was the Wrap-up week (mean 53.03 [SD] 37.91), and the one with the lowest was the Welcome week (mean 25.13 [SD] 22.34). Figure 1 presents the full distribution of participants meeting respective adherence criteria across 1-8 modules.

### Preliminary Analyses

#### Assessment of Missing Data

Results of independent *t* tests and chi-square tests treating posttest data missing status as the independent variable in relation to other variables revealed that only condition and program initiation were significantly related to posttest data missing status (*P*<.001). By contrast, there were no significant differences between posttest data missing status and GRIT, TMQ, and PHQ-9 (*P*=.81-.08). The Little MCAR test confirmed that our data set was not MCAR and therefore utilizing the listwise deletion would result in biased coefficients (χ^2^_38_=399.95, *P*<.001). For this reason, multiple imputation was used instead to handle missing data in our subsequent linear regression models.

#### Covariate Identification Analyses

Results of independent *t* tests and chi-square tests revealed that suicidal ideation was significantly related to DASS-21 change scores (*P*<.001). Results also revealed that GRIT, TMQ, PHQ-9, SHS, program initiation, gender, and condition were not significantly related to DASS-21 change scores (*P*=.07-.9). However, gender had been a significant covariate in the main RCT analyses [16], and the condition variable is conceptually meaningful, given that our data set collapsed pre-post outcomes from each condition at different respective assessment periods. Therefore, suicidal ideation, gender, and condition were entered as covariates in our regression models.

#### Assumption Checks

We conducted assumption checks to confirm utilizing multiple linear regressions was appropriate [24]. The following assumptions were met: (1) 1 continuous dependent variable; (2) multiple independent variables; (3) independence of observations (Durbin-Watson=1.99); (4) linearity between the dependent and independent variables (confirmed via separate scatter plots); and (5) homoscedasticity (confirmed via residual scatter plots based on a model including all adherence variables and covariates). However, assumption 6 requiring that data must not show multicollinearity between all variables was not met (variance inflation factor=1.00-12.77). Therefore, moving forward we tested each independent variable in its own model (including covariates), and there was no multicollinearity (variance inflation factor=1.00-1.01). We also identified outliers and potential influential cases through examination of models’ scatter plots, leverage, Cook *D*, and DFBETA values. Although some participants exceeded respective diagnostic test values, DFBETAs revealed little influence specifically from our adherence variables of interest. Specifically, only 2 participants were influential on 1 adherence variable, with no participants identified as influential for the remaining 5 adherence variables. Therefore, no cases were removed from subsequent analyses. Assumption 8 requiring normally distributed residuals was confirmed by graphs of each model’s standardized residuals: histograms looked approximately normal and each Q-Q plot showed a linear line.

### Main Findings (Intent to Treat)

See Table 2 for results of all 6 regressions. Linear regressions revealed that high behavioral adherence significantly predicted symptom improvement (unstandardized *B*=–0.26, *P*=.04). Additionally, moderate attitudinal adherence also significantly predicted symptom improvement (unstandardized *B*=–0.36, *P*=.03). All other measures of behavioral and attitudinal adherence did not significantly predict symptom improvement (*P*=.05-.10). None of the observed *P* values from these 6 regressions (*P*=.03-.10) fell below their respective Benjamini-Hochberg critical value (0.008-0.50), indicating that significant results may be due to false discovery rate.

**Table 2:** Results from linear regressions examining 6 measures of adherence.

Adherence term in model and variables			Intent-to-treat sample (n=947)					
		Pooled *R^2^*		Pooled *B*		*P* value		
**Behavioral minimal effort**			0.068					
	Gender			–7.21		.77		
	Condition			–0.26		.17		
	SI^a^			–1.03		<.001		
	Adherence			–0.24		.05		
**Behavioral moderate effort**			0.067					
	Gender			–7.18		.73		
	Condition			–0.31		.16		
	SI			–0.99		<.001		
	Adherence			–0.22		.07		
**Behavioral high effort**			0.067					
	Gender			–0.33		.70		
	Condition			–0.99		.16		
	SI			–7.16		<.001		
	Adherence			–0.26		.04		
**Attitudinal minimal effort**			0.067					
	Gender			–0.28		.74		
	Condition			–0.94		.19		
	SI			–7.20		<.001		
	Adherence			–0.23		.07		
**Attitudinal moderate effort**			0.069					
	Gender			–0.32		.71		
	Condition			–1.00		.16		
	SI			–7.11		<.001		
	Adherence			–0.36		.03		
**Attitudinal high effort**			0.065					
	Gender			–0.38		.66		
	Condition			–0.96		.18		
	SI			–7.04		<.001		
	Adherence			–0.51		.10		

^a^SI: suicidal ideation.

### Post Hoc Findings (Initiators-Only Sample)

As a post hoc check, we reran the main analyses excluding those that did not initiate any activity, for quantitative and conceptual reasons. Quantitatively, the distribution plots of the adherence variables reveal that data are largely skewed to the left (Figure 1), which is largely due to 21.1% (200/947) of participants not initiating any type of activity on the platform. Additionally, preliminary *t* tests revealed that program initiation was significantly related to postdata missingness (*t*_945_*=*–9.29, *P*<.001). Conceptually, previous research has shown that despite a large number of individuals enrolling in self-guided online interventions, very few actually initiate the program [9]. For example, a systematic review of self-guided online interventions for depression and anxiety found that 33%-88% of users who downloaded an app actually used it at least once [2]. Thus, we expected that an initiator sample might reveal different findings about the relationship between adherence and outcomes for the self-guided online program.

The initiator sample consisted of 747 students, with a mean age of 23.01 (SD 5.56), who were 78.8% (n=589) female, 31.3% (n=234) White, and 11.0% (n=82) international. At baseline, the mean DASS-21 total score was 17.55 (SD 10.59). In this sample, the number of people with post-DASS-21 data was 410 (54.9%), indicating this subsample had more complete pre-post data than the full sample. The *t* and chi-square tests examining the differences between program initiators and noninitiators revealed that gender was significantly related to program initiation, such that female students were more likely to initiate than male students (*P*=.01). All other variables (suicidal ideation, *P*=.21; TMQ, *P*=.18; GRIT, *P*=.46; PHQ-9, *P*=.07; and SHS, *P*=.98) were not significantly related to program initiation. Linear regressions revealed that none of the adherence terms in all 6 linear regressions models significantly predicted symptom improvement (*P*=.28-.08).

## Discussion

### Summary of Findings

Given that online interventions offer users ease of accessibility, autonomy, and flexibility, many researchers are greatly interested in identifying indicators of adherence to online interventions that are predictive of symptom improvement. Because of the lack of consensus in the literature regarding the operationalization and measurement of adherence, our first aim was to demonstrate a process for intentionally defining multiple measures of adherence to test their utility in predicting symptom improvement. To achieve this aim, we created multiple measures that fit into universal categories (eg, behavioral, attitudinal) but that were still intervention specific (eg, number of skill practice logs, word count on weekly check-ins). Each adherence measure was specified based on the interventions’ intended use (eg, 3 levels of effort based on the platform’s virtual medal system). We believe that “behavioral” and “attitudinal” dimensions of engagement are universal enough to be widely applicable across intervention designs, allowing for comparison between interventions, though the intervention-specific adherence metrics may still vary. For example, these categories could be applied to Headspace [27], a popular meditation online application. In this case, behavioral adherence could be defined as the number of meditation modules used or the number of minutes listed; attitudinal adherence could be defined as completing the check-in questions in the “Journey” section or customizing the notifications in the “Settings” section.

Results from the intent-to-treat sample demonstrated that behavioral and attitudinal measures of adherence were predictive of symptom improvement at differing levels of effort. Specifically, high-effort behavioral adherence (ie, number of modules with at least five logs; *P*=.04) and moderate-effort attitudinal adherence (ie, number of modules with check-in word count at or above respective average; *P*=.03) predicted significantly more decrease in DASS-21 total scores. By contrast, results from the initiator sample revealed none of the adherence measures as predictive of symptom improvement. In other words, when our adherence measures were tested with a more conservative definition of the intervention user sample, the previously detected adherence effects disappeared. In summary, whether or not adherence effects surmounted conventional levels of statistical significance (ie, 5% probability of being observed due to random chance) depended on definitions of *how* the intervention is used (ie, type of adherence), *how much* it is used (ie, effort of adherence), and also *who* is a user (ie, intervention sample criteria).

### Implications

Although the aggregate of our findings could be interpreted as weak evidence for adherence effects within self-guided online mental health interventions, we instead interpret them as supporting just how challenging it is to measure such dose-response effects. If such adherence were truly unimportant, then we would have expected larger *P* values for most or all adherence terms (Table 2). By contrast, our 6 main models converged such that the negative relationship between intervention adherence and symptoms had only a 3%-10% probability of being observed due to random chance. Regarding the relatively low amount of variance explained, it is likely due at least in part to statistical constraints. On the one hand, we are measuring an extremely diverse independent variable: the seemingly infinite ways and degrees that individuals can adhere to self-guided online interventions [10]. One the other hand, we are simultaneously trying to detect changes in a constrained dependent variable: self-guided online mental health interventions generally produce smaller effects with a restricted range of improvement [4,28]. Indeed, the main trial results for the currently tested intervention found robust but small effects [16]. This dilemma can be characterized as testing high-variance dose (ie, adherence) in the prediction of small-effects response. Unfortunately, we are still left with the unresolved questions of *which* and *how much* adherence should be prioritized for users of self-guided online interventions. In service of resolving these questions in the future, we have translated some lessons learned into recommendations that future researchers and developers could use when investigating the role of adherence within a specific online intervention (Table 3). We hope that these recommendations will assist others in understanding and measuring adherence in a more thorough and standardized manner. We elaborate below on some potential additional benefits that following our outlined recommendations could provide.

**Table 3:** Recommendations for research on adherence-outcome effects of online interventions.

Item	Recommendation	Section cross-reference
Operationalize adherence measures	Literature review: Identify relevant research definitions of adherence and recommendations for developing adherence definitions/measures. Given that measures of adherence on guided or self-guided online interventions are variably defined in the current literature, it is important to use past knowledge to move toward standardization. Define multiple measures: Based on that review, create multiple measures of adherence to the given online intervention. Because of the highly variable designs and features of online interventions, adherence to them can be measured in many ways. Defining multiple measures a priori and reporting on all of them allow for testing of differential effects of adherence (ie, to which features? How much?) on improvement in outcomes.	See the “Introduction,” “Adherence,” “Behavioral Adherence,” and “Attitudinal Adherence” sections for relevant literature. See the “Adherence” section for results supporting variability in respective adherence rates. See the “Main Findings (Intent to Treat)” section for main results demonstrating significance tests varying for each adherence term.
Select primary outcome measure	Carefully select your outcome measure with attention to maximizing detection of adherence-outcome effects. Given that online interventions are prone to small main intervention effects, the sensitivity of an outcome measure is especially crucial for adherence-outcome effects. Note: If you have multiple outcome measures or multiple subscales, you might maximize your power by selecting that which showed largest effect size per your primary intervention main effect analyses.	See the “Primary Outcome” section for methodological support of our choice of primary outcome, based on previously reported results. See Table 2 for demonstration of small effects.
Select an appropriate data analytic plan	Model selection: Design an analysis plan that is appropriate for the specific goal of testing adherence-outcome effects. For example, because users can be simultaneously adhering to multiple aspects of an online intervention, it is all the more important to check for collinearity. If such assumptions would be violated, adherence measures must be separately tested as predictors. Covariates selection: Select covariates with primary intervention analyses in mind. Because adherence-outcome effects are presumably tested after primary intervention effects, covariation selection should be consistent across both.	See the “Preliminary Analyses” and “Linear Regressions” sections for justification of model selection. See the “Assumption Checks” section for model assumption check results. See the “Covariates” section for justification of covariate selection. See the “Covariate Identification Analyses” section for results supporting our covariate selection.
Identify method to deal with missing data	First, identify the rate of missingness in your primary outcome measure. Once a rate is identified, select an appropriate method for dealing with missing data (ie, last observation carried forward, raw data, completer only). Because online interventions often experience high dropout, a larger proportion of data may be missing, and thus results could drastically change by imputation decision.	See the “Multiple Imputation” section for methodological rationale. See the “Assessment of Missing Data” section for results supporting choice of imputation method.
Define your sample	Defining your intent-to-treat and initiator sample can be less clear-cut for online intervention research. Study enrollment does not guarantee a user has completed intervention enrollment (eg, created an account, downloaded the app). Furthermore, enrollment in the online intervention does not guarantee intervention initiation (eg, signing in to read content at least once, completing at least one practice activity). Thus, for studies investigating adherence to online interventions, it is important to consider which sample had true measurement of adherence (eg, versus failure to complete intervention enrollment).	See the “Participants” section for methodological justification. See the “Assessment of Missing Data” section for results demonstrating different adherence dose-response results for intent-to-treat versus initiator-only samples.
Report all your results	Ensure that all your findings regarding each adherence measure are clearly reported in your paper. Because there is such much variability in research findings about online intervention adherence-outcome effects, knowing null results will help future researchers and intervention developers better disentangle where adherence matters most.	See the “Results” section and Table 2 for thorough reporting of preliminary, main, and post hoc analyses.

Testing multiple measures of adherence is critical for understanding dose-response effects across diverse users. According to Sieverink and colleagues [11], there appears to be an assumption in the literature that a user must interact with all aspects of the intervention to benefit. As such, researchers often define and operationalize adherence based on this assumption. However, online interventions are highly variable in their designs, components, instructions, and goals. Indeed, our results support the idea that users can adhere strongly to some aspects of an intervention, but not adhere to others. If we had assumed otherwise in this study, we would not have been able to see that multiple types of adherence at varying levels of effort could be associated with symptom improvement in our intent-to-treat sample. Therefore, perhaps there will never be a single answer about *which* type of adherence matters; rather, researchers should continue testing multiple adherence measures to better understand dose-response effects for the wide audience of self-guided online intervention users.

Our study also demonstrated that discrepant adherence results can arise depending on how the “user” sample is determined. When all individuals who created an account (ie, intent-to-treat sample) were included in the sample, results revealed that relatively higher levels of effort (ie, high behavioral adherence and moderate attitudinal adherence) predicted symptom improvement. When only those individuals with account activity within the intervention (ie, program initiators) were included, none of the adherence measures were predictive of symptom improvement. Unfortunately, the differentiation between study enrollment, intervention enrollment, and intervention initiation is rarely reported in online intervention trials. To use 2 specific trials as examples, Donkin et al [15] included participants based on study enrollment (ie, those who completed a 3-month follow-up assessment), whereas Christensen et al [29] included participants based on intervention initiation (ie, those who completed at least one activity in the intervention). Yet the difference in study enrollment rates versus intervention initiation rates may be especially pronounced in the context of open-source online interventions, given the ease of access to signing up (compared with face-to-face interventions). Indeed, as previously mentioned, a systematic review of self-guided online interventions found that 33%-88% of users who downloaded an app actually used it at least once [2]. Knowing that attrition and sporadic use are to be expected in the context of online interventions [9], we strongly recommend that researchers both (1) clearly report and justify how they defined their “user” sample, and (2) report results based on different user sample types.

### Limitations

The main limitation in this study, which is expected in the context of online interventions, is the large proportion of missing postintervention data. To ameliorate this, we used multiple imputations, and generated a large number of data sets while including any potential auxiliary variables to support model generation of estimates. As compared with other methods of dealing with missing data such as listwise and pairwise deletion, multiple imputation is considered a “state-of-the-art” technique and is a recommended procedure in the methodological literature [30]. However, the inability to confirm that the data set was missing at random could have affected the interpretation of our results. Next, detection of any significant adherence-outcome effects was difficult given the overall small intervention effects, per our small R^2^ values. Such small effects are unfortunately inherent to research on an online prevention program (see [16]), and there is also a prohibitive “floor effect” when a nonclinical sample can only improve so much. Another main limitation to the study is the generalizability of our results. There may be more efficient and accurate ways of measuring adherence to interventions for substance use disorders, for example, or for younger or older populations. Replication of findings will be important. An additional limitation in our study was our use of word count to operationalize attitudinal adherence, which has not been a previously established way of defining attitudinal adherence. Word count was selected as an indicator in this study given the high feasibility and ease of collection. However, future research is needed to validate word count as a measure of attitudinal adherence. Specifically, researchers might consider qualitative coding to identify themes associated with attitudinal adherence and examine correlation with word count.

### Conclusion

First and foremost, researchers are encouraged to use the checklist in Table 3 as a resource for recommendations when planning studies, and to report any further decisions pertinent to examining adherence to online interventions. Researchers are also encouraged to reproduce and expand the behavioral and attitudinal categories that we have outlined. Although results of this study provide some preliminary evidence for the predictive validity, additional research is needed to determine the generalizability of these candidates. Finally, researchers are also encouraged to examine individual characteristics (eg, personal traits, baseline symptom severity, technology preference) that may moderate the relationship between adherence and outcome. Such findings could be used to inform customization of interventions to maximize benefit based on relevant personal characteristics.

In conclusion, online interventions offer its users the autonomy to interact with the platform, resulting in a variety of ways that individuals could adhere to the intervention. This poses a challenge for researchers who aim to understand the role of adherence in improving these interventions. However, through the accumulation of high-quality and transparent research into the numerous forms of adherence that result in symptom change, it will be possible to prioritize features and make design decisions to maximize effectiveness of online interventions. With this end goal in mind, this paper does not claim to have identified the best way to measure adherence, rather we add to the ongoing discussion by summarizing our lessons learned to facilitate this discussion and the process of defining and measuring adherence. By creating a more efficient and standardized process for future researchers, we hope to facilitate the creation of high-quality transparent research to understand the role of adherence in self-guided online interventions.

## Abbreviations

DASS-21: 21-item Depression, Anxiety, and Stress Scale
GRIT: Grit Scale
MCAR: missing completely at random
PHQ-9: 9-item Patient Health Questionnaire
RCT: randomized controlled trial
SHS: Subjective Happiness Scale
TMQ: Treatment Motivation Questionnaire
